# Editorial for the Special Issue on Passive Micromixers

**DOI:** 10.3390/mi9050250

**Published:** 2018-05-21

**Authors:** Arshad Afzal, Mubashshir A. Ansari, Kwang-Yong Kim

**Affiliations:** 1 Department of Mechanical Engineering, Indian Institute of Technology Kanpur, Kanpur, Uttar Pradesh 208016, India; aafzal@iitk.ac.in; 2 Department of Mechanical Engineering, Aligarh Muslim University, Aligarh, Uttar Pradesh 202001, India; mub.ansari@yahoo.com; 3 Department of Mechanical Engineering, Inha University, Incheon 22212, Korea

Micromixers are important components of microfluidic systems, such as the lab-on-a-chip and micro-total analysis system (μ-TAS) designed for a large number of applications, including sample preparation and analysis, drug delivery, fast chemical reactions, and biological and chemical synthesis. Typically, micromixers operate from a very low to high Reynolds number range of 0.001–1000 in the laminar flow regime. Therefore, the random turbulent motions that are necessary to homogenize fluid samples are absent at the microscale. As a result, the mixing processes inside these microfluidic systems are diffusion-dominated and slow in addition to requiring a long channel length. Therefore, efficient mixing over a short channel length is a challenging task. Micromixers can be broadly classified as active (based on external energy/stimulus) and passive (based on geometrical modifications) micromixers. Among the two basic concepts, the passive micromixers do not rely on external mechanisms and offer the advantages of simple design and easy fabrication. This special issue focuses on presenting the development of efficient and robust designs of passive micromixers. 

A total of 10 original research papers, which cover important aspects of micromixing technology, are published in the special issue. The important areas being focused upon are the numerical and experimental analyses of flow and mixing in different micromixers [1,2,3,4,5], optimization [6,7,8], and fabrication of micromixers [9]. In the review paper [10], recent developments in both active and passive micromixers are summarized and discussed. 

Computational fluid dynamics (CFD) analysis of flow and mixing is seen as an important tool in designing passive micromixers in eight research articles [1,2,3,4,5,6,7,8]. Javaid et al. [1] proposed a serpentine-shaped micromixer with sinusoidal walls and conducted a CFD analysis using COMSOL Multiphysics software. The numerical results showed a good agreement with a previously published experiment, with improved mixing performance compared to a simple serpentine channel. A similar investigation of flow and mixing in serpentine channels with a non-rectangular cross-section was conducted by Clark et al. [2]. The results indicate enhancement in mixing performance using non-rectangular cross-section serpentine micromixers. Ansari et al. [3] performed both numerical and experimental analyses of a vortex micro T-mixer using ANSYS-CFX. The design is promising as it can efficiently increase the mixing for T-junction micromixers. Wang et al. [4] carried out CFD analysis of a herringbone passive micromixer for pressure-driven and electro-osmotic flow. A good mixing performance was observed at low flow rates for both flow situations. However, at a fixed flow rate, the mixing performance is superior in the case of electro-osmotic flow than pressure-driven flow. Okuducu and Aral [5] conducted a different study, which focused on the comparative evaluation of the finite volume method (FVM) and finite element method (FEM) to characterize numerical errors in mixing analysis. Using T-mixer geometry and two CFD tools, which were namely OpenFoam (FVM-based) and COMSOL Multiphysics (FEM-based), a detailed analysis based on mesh structure, orientation and flow parameters was presented. Using CFD and Taguchi statistical method, Solehati et al. [6] studied a T-junction micromixer with wavy walls to evaluate the effect of key design parameters on the mixing performance, pumping power and figure of merit (mixing index per unit pressure drop). The Taguchi method was found to be robust in determining the optimum combination of the design parameters. Raza et al. [7] carried out multi-objective optimization of a serpentine micromixer with crossing channels at low and high Reynolds numbers. Pareto-optimal fronts representing the trade-off between conflicting objectives, mixing index and pressure drop were obtained for both low and high Reynolds numbers. Guo et al. [8] demonstrated topology optimization based on Lagrangian mapping method for mixing problems that were dominated completely by the convection with negligible diffusion. The layout of the passive micromixers was determined by solving a topology optimization problem to minimize the mixing measurement. 

Shan et al. [9] fabricated two types of micromixers with complex structures using the femtosecond laser wet etch (FMWE) technology inside fused silica. Using FMWE technology, the multi-microchannel mixers with high integration and uniformity for high-performance applications were realized, demonstrating the flexibility and universality of FLWE technology. Finally, the review paper by Cai et al. [10] summarized the recent advances in passive and active micromixers for microfluidic applications. In recently published articles, active micromixers have used pressure fields, electrical fields, acoustics, magnetic fields, and thermal fields as external energy sources to perturb the fluid. Furthermore, passive micromixers based on geometrical modifications using two-dimensional obstacles, unbalanced collisions, convergence–divergence structures or three-dimensional lamination and spiral structures were discussed. 

We would like to take this opportunity to express our appreciation to all the authors for submitting their papers and contributing to the success of this special issue. We also want to thank all the reviewers for dedicating their time and helping to improve the quality of the submitted papers and professional staff at the Micromachines Editorial office for providing invaluable assistance.
